# Exploring the genetic diversity of genotypes G8 and G10 of the *Echinococcus canadensis* cluster in Europe based on complete mitochondrial genomes (13 550–13 552 bp)

**DOI:** 10.1017/S0031182023000331

**Published:** 2023-06

**Authors:** Teivi Laurimäe, Liina Kinkar, Epp Moks, Guna Bagrade, Urmas Saarma

**Affiliations:** 1 Department of Zoology, Institute of Ecology and Earth Sciences, University of Tartu, Juhan Liivi 2, 50409 Tartu, Estonia; 2 Estonian Veterinary and Food Laboratory, Kreutzwaldi 30, 51006 Tartu, Estonia; 3 Latvian State Forest Research Institute ‘Silava’, 111 Rigas str., LV-2169 Salaspils, Latvia

**Keywords:** cystic echinococcosis, *Echinococcus canadensis*, genotype G8, genotype G10, mitochondrial DNA

## Abstract

*Echinococcus granulosus sensu lato* is a group of tapeworm species known to cause cystic echinococcosis. Within this group, the *Echinococcus canadensis* cluster includes genotypes G8 and G10 that have a predominantly sylvatic life cycle – transmission occurs between wild cervids and wolves. Relatively few studies have explored the genetic variation of the elusive G8 and G10, and their extent of genetic variation is yet to be investigated at the complete mitochondrial (mt) genome level. The aim was to explore the genetic variation of these 2 genotypes in Europe using complete mtDNA sequences and provide a high-quality reference dataset for future studies. Sequences of complete mt genomes were produced for 29 samples of genotype G8 and G10 from wolves, moose, reindeer and roe deer, originating from Finland, Sweden, Russia, Poland, Latvia and Estonia. Genetic variation was explored based on phylogenetic network analysis, revealing marked differences between G8 and G10 (over 400 mutations), and more detailed patterns of variability within the 2 genotypes than previously observed. Understanding the mt genetic composition of a species provides a baseline for future studies aiming to understand whether this mt distinctiveness is mirrored in the nuclear genome and whether it has any impact on any phenotypic traits or parasite transmission.

## Graphical abstract

**Figure:**
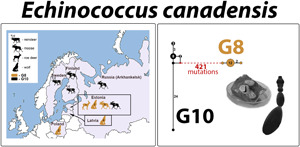

## Introduction

The *Echinococcus canadensis* cluster (comprising several genotypes) belongs to a group of cestode species called *Echinococcus granulosus sensu lato* (*s.l.*) (Vuitton *et al*., 2020). These are tapeworms that cause cystic echinococcosis (CE) in humans – a disease recognized by the World Health Organization as a neglected tropical disease (World Health Organization, 2021). Within the *E. canadensis* cluster, the genotypes G8 and G10 are predominantly maintained in a sylvatic transmission cycle, involving wolves (*Canis lupus*) as definitive hosts harbouring adult worms, and wild cervids such as moose (*Alces alces*), elk/wapiti (*Cervus canadensis*) and roe deer (*Capreolus capreolus*) as intermediate hosts harbouring larvae (Marcinkuté *et al*., 2015; Romig *et al*., 2017). Synanthropic transmission cycle involving semi-domesticated reindeer (*Rangifer tarandus*) and dogs, and a semi-synanthropic life cycle involving wild cervids and free-roaming and/or hunting dogs are also known to occur (Oksanen and Lavikainen, 2015; Romig *et al*., 2017). The larval stage of this parasite represents a fluid-filled cyst predominantly found in the liver of the intermediate hosts. However, the available evidence suggests *E. canadensis* (G8/G10) is more prone to establish infection in the lungs, with a more benign CE pathology than CE infections caused by other *Echinococcus* spp. (reviewed in Oksanen and Lavikainen, 2015). Protoscoleces proliferate within the cyst and subsequently develop into adult worms in the small intestine of the definitive hosts upon ingestion of infected internal organs of the intermediate hosts (Thompson, 2017). Humans are considered as accidental intermediate hosts and represent a ‘dead-end’ for the parasite (Kern *et al*., 2017). Although the exact pathways of infection are not yet fully understood, it is thought the infection typically occurs through ingestion of food or water that are contaminated with *Echinococcus* eggs (Alvarez Rojas *et al*., 2018).

According to mitochondrial (mt) cytochrome c oxidase subunit 1 (*cox*1; 366 bp) and NADH dehydrogenase subunit 1 (*nad*1; 474 bp) gene fragments, 10 genotypes, i.e. G1–G10 (formerly known as strains), were originally defined within *E. granulosus s.l.* (Bowles *et al*., 1992, 1994; Bowles and McManus, 1993; Lavikainen *et al*., 2003). A number of these genotypes have now been classified as distinct species within the cluster and two genotypes are considered as invalid (G2 and G9) (Kedra *et al*., 1999; Kinkar *et al*., 2017; reviewed in Lymbery, 2017). The species status of genotypes G6/G7 (formerly camel and pig strains, respectively) and G8/G10 (cervid strains) has, however, remained unclear. The mtDNA data show G10 to be more closely related to G6/G7 than to G8 (e.g. Nakao *et al*., 2006; Moks *et al*., 2008; Knapp *et al*., 2011), while evidence from nuclear genes has indicated that the cervid G8 and G10 form one clade, and G6/G7 another (Saarma *et al*., 2009; Laurimäe *et al*., 2018*a*). As such, some have suggested that G8/G10 should be regarded as one species (*E. canadensis*) together with G6/G7 (e.g. Nakao *et al*., 2006), while others have proposed three (Lymbery *et al*., 2015) or two species (Thompson, 2008; Saarma *et al*., 2009; Laurimäe *et al*., 2018*a*). Until the taxonomic dispute has been resolved, the authors have herein elected to refer to genotypes G6–G8 and G10 as genotypes of the *E. canadensis* cluster or whenever *E. canadensis* is mentioned, genotypes are also specified (Vuitton *et al*., 2020).

The distribution range of *E. canadensis* (G8/G10) is circumpolar and limited to North America (Canada; Alaska, USA), Fennoscandia (Finland; Sweden), continental north-eastern Europe (Estonia, Latvia) and Russia (Yakutia, Siberia) (reviewed in Oksanen and Lavikainen, 2015 and Romig *et al*., 2017). The prevalence of *E. canadensis* (G8/G10) in European wildlife has been documented rather sporadically, while human infections are considered rare but have likely been underreported (reviewed in Marcinkuté *et al*., 2015; Oksanen and Lavikainen, 2015, Davidson *et al*., 2016; Deplazes *et al*., 2017; Casulli *et al*., 2022*a*, 2022*b*). To the best of our knowledge, the only published case in Europe of human infection with confirmed *E. canadensis* G10 was recorded in Finland (Hämäläinen *et al*., 2015). Although in other countries e.g. in Estonia and Latvia where *E. canadensis* (G8/G10) is known to be present in wildlife, human CE infections are known to occur, but are not molecularly characterized. Data on *E. canadensis* (G8/G10) prevalence in wild mammals in Europe have shown that e.g. 0.8% of moose (16 of 2038) and 3.8% of wolves (1 of 26) in Estonia harbour *E. canadensis* (G8/G10; Moks *et al*., 2006, 2008), while in Latvia the parasite has been detected in 2.9% of wolves (1 of 34) (Bagrade *et al*., 2009), and in Finland in 10% of wolves (during 2000–2010, 25 of 252) (reviewed in Oksanen and Lavikainen, 2015).

To date, studies exploring the genetic diversity of genotypes G8 and G10 have been scarce. This is likely due to difficulties in obtaining parasite samples from wildlife species (wolves, wild cervids), particularly as surveillance of the parasite infection in wildlife is not regularly performed and the detection and collection of *Echinococcus* spp. parasite tissue are laborious tasks (Moks *et al*., 2008; Schurer *et al*., 2013; Romig *et al*., 2017). However, by analysing selected mt loci, predominantly *cox*1 and/or *nad*1 gene fragments, available studies have provided valuable first insight into the genetic variation of these genotypes (e.g. Lavikainen *et al*., 2006; Moks *et al*., 2008; Nakao *et al*., 2013; Schurer *et al*., 2013; Yang *et al*., 2015; Dell *et al*., 2020; Priest *et al*., 2021). For example, such studies have indicated that the extent of genetic variation within the two *E. canadensis* genotypes (G8 and G10) is considerably lower and less complex than within other *Echinococcus* species or genotypes, such as *E. granulosus sensu stricto* (*s.s*., G1/G3) and G6/G7 of the *E. canadensis* cluster (Romig *et al*., 2015; Kinkar *et al*., 2018*a*, 2018*b*; Laurimäe *et al*., 2018*b*).

The aim of the current study was to explore the extent of genetic variation based on complete mtDNA sequence data of *E. canadensis* (G8 and G10) specimens originating from the main distribution range of the parasite in Europe and provide a high-quality reference dataset for future genetic studies.

## Materials and methods

### Biological material

**Figure 1.**
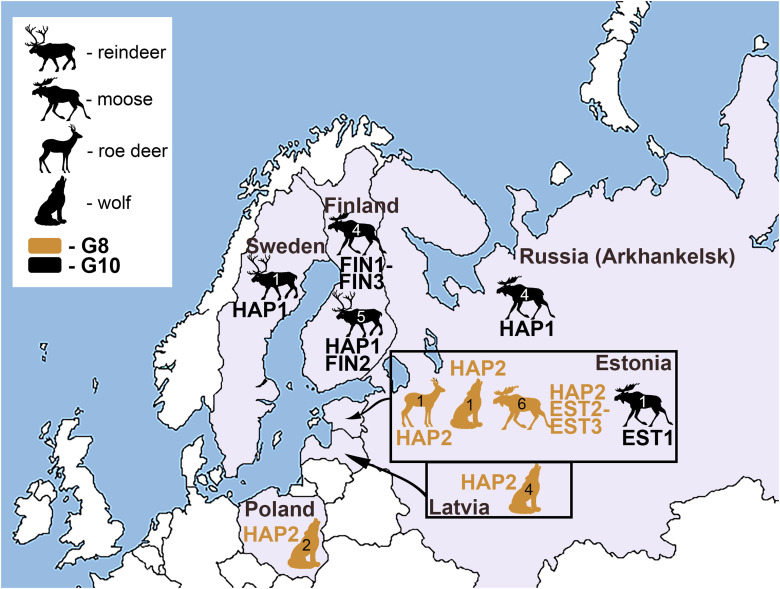
Map of sampling locations for genotypes G8 and G10 of the *Echinococcus canadensis* cluster, and their respective host species. Samples designated as genotype G8 are represented by light brown colour, and G10 by black. A number inside the silhouette of a host species represents the number of parasite specimens analysed (a single cyst or adult worm per host animal). Three-letter abbreviations represent the names of haplotypes based on complete mt genome sequences.

A single *Echinococcus* cyst or adult worm per host animal was analysed. *Echinococcus* cyst samples (*n* = 22) were obtained from three intermediate host species (moose, *n* = 15; reindeer, *n* = 6; roe deer, *n* = 1) from four countries (Sweden, Finland, Russia and Estonia), whereas adult worms were collected from a definitive host species (wolf, *n* = 7) from three countries (Estonia, Latvia and Poland) (Fig. 1; Table 1). The samples were not collected specifically for the purposes of the current study – cyst material or adult worms were obtained during routine inspections at slaughter or inspection of hunted animal carcasses during hunting season and were donated to the University of Tartu (Estonia). All biological material was stored at −20°C until further analysis.

**Table 1.** Identity and origin of *Echinococcus canadensis* G8 and G10 specimens analysed in the current study

GenBank accession	Sample ID	Haplotype	Host animal	Country	Genotype
OQ161094	Sample 1	HAP2	Wolf	Latvia	G8
OQ161095	Sample 2	HAP2	Wolf	Latvia	G8
OQ161096	Sample 3	HAP2	Wolf	Latvia	G8
OQ161097	Sample 4	HAP2	Wolf	Latvia	G8
OQ161098	Sample 5	HAP2	Wolf	Poland	G8
OQ161099	Sample 6	HAP2	Wolf	Poland	G8
OQ161100	Sample 7	HAP2	Wolf	Estonia	G8
OQ161101	Sample 8	HAP2	Roe deer	Estonia	G8
OQ161102	Sample 9	HAP2	Moose	Estonia	G8
OQ161103	Sample 10	HAP2	Moose	Estonia	G8
OQ161104	Sample 11	EST2	Moose	Estonia	G8
OQ161105	Sample 12	HAP2	Moose	Estonia	G8
OQ161106	Sample 13	EST3	Moose	Estonia	G8
OQ161107	Sample 14	HAP2	Moose	Estonia	G8
OQ161108	Sample 15	EST1	Moose	Estonia	G10
OQ161109	Sample 16	FIN1	Moose	Finland	G10
OQ161110	Sample 17	FIN2	Moose	Finland	G10
OQ161111	Sample 18	FIN3	Moose	Finland	G10
OQ161112	Sample 19	FIN3	Moose	Finland	G10
OQ161113	Sample 20	FIN2	Reindeer	Finland	G10
OQ161114	Sample 21	FIN2	Reindeer	Finland	G10
OQ161115	Sample 22	HAP1	Reindeer	Finland	G10
OQ161116	Sample 23	HAP1	Reindeer	Finland	G10
OQ161117	Sample 24	HAP1	Reindeer	Finland	G10
OQ161118	Sample 25	HAP1	Moose	Russia (Arkhangelsk region)	G10
OQ161119	Sample 26	HAP1	Moose	Russia (Arkhangelsk region)	G10
OQ161120	Sample 27	HAP1	Moose	Russia (Arkhangelsk region)	G10
OQ161121	Sample 28	HAP1	Moose	Russia (Arkhangelsk region)	G10
OQ161122	Sample 29	HAP1	Reindeer	Sweden	G10

### Sample preparation, polymerase chain reaction (PCR) and sequencing

Genomic DNA was extracted from parasite material using a High Pure PCR Template Preparation Kit (Roche Diagnostics, Mannheim, Germany), according to the manufacturer's instructions. PCRs and amplification were carried out as described in Laurimäe *et al*. (2018*b*). Briefly, 13 primer pairs were utilized to amplify the complete mt genome (~13 500 bp) of the parasite. Each PCR was carried out in a volume of 20 *μ*L, with 0.25 *μ*m of each primer, 1× BD Advantage 2 PCR buffer (BD Biosciences, Franklin Lakes, NJ, USA), 0.2 mm dNTP (Fermentas, Vilnius, Lithuania), 1 U Advantage 2 Polymerase mix (BD Biosciences) and <1 *μ*g of template DNA. Touchdown PCRs were carried out as described in Laurimäe *et al*. (2018*b*), with initial denaturation at 95°C for 1 min, followed by 10 cycles of 95°C for 20 s, 55°C for 45 s (annealing temperature progressively reduced by −0.5°C in each cycle) and 68°C for 2 min; followed by 25 cycles of 95°C for 20 s, 50°C for 45 s, 68°C for 2 min; and finishing with a final elongation step at 68°C for 3 min. Of the 20 *μ*L, 10 *μ*L of the PCR products were examined on a 1.2% agarose gel. The remaining 10 *μ*L was subjected to purification with 1 U shrimp alkaline phosphatase and 1 U exonuclease I (both from Thermo Scientific, Waltham, USA), and subsequent incubation at 37°C for 30 min, followed by 80°C for 15 min in order to inactivate the enzymes. Sequencing was performed at the Core Facility of Genomics (Tartu, Estonia) using the same set of primers as for the initial PCR. Both forward and reverse strands were sequenced.

### Sequence assembly, quality control and alignment

Consensus sequences were assembled in Codon Code Aligner v.6.0.2 and each polymorphic position was verified by eye using ‘raw’ chromatogram data. Sequence alignment using Clustal W was performed in BioEdit v.7.2.5 (Thompson *et al*., 1994; Hall, 1999). An initial assessment of the genotypic identity of the samples was performed using NCBI Nucleotide BLAST.

### Phylogenetic analysis

First, complete mt genome sequences of other closely related *E. granulosus s.l.* species were retrieved from the GenBank database (Table S1) and aligned with the sequences produced in this study, to assess the extent of genetic variation between and among distinct genotypes (datasets A and B, respectively). For this, median-joining phylogenetic networks were constructed using Network v.4.6.1.6 (Fluxus Technology Ltd; Colchester, UK.) software, with both indels and point mutations considered. As the commonly applied mtDNA markers had been developed at a time when the extent of mtDNA variation was largely unknown (particularly for G8 and G10), we also aimed to assess and compare the degree of inter- and intra-genotypic phylogenetic resolution provided by other commonly used markers (i.e. *cox*1, *nad*1 and 12S rRNA; e.g. Bowles *et al*., 1992; Bowles and McManus, 1993; Trachsel *et al*., 2007). Outgroups, i.e. other species/genotypes that served as reference groups, were included as required (accession numbers are listed in Table S1). Phylogenetic networks were constructed as described above.

## Results

### Genotypic identity and origin of analysed samples

All 29 samples analysed here were identified as *E. canadensis* genotype G8 (*n* = 14) or G10 (*n* = 15). For each specimen, the complete mt genome sequence was produced (13 550–13 552 bp). All sequences were deposited in the GenBank database (accession numbers OQ161094–OQ161122; Table 1). The G8 samples that were successfully sequenced originated from Estonia (roe deer, *n* = 1; wolf, *n* = 1; moose, *n* = 6), Latvia (wolves, *n* = 4) and Poland (wolves, *n* = 4). The G10 samples were from Estonia (moose, *n* = 1), Finland (moose, *n* = 4; semi-domesticated reindeer, *n* = 5), Sweden (semi-domesticated reindeer, *n* = 1) and Russia (moose, *n* = 4).

### High genetic variability between *Echinococcus* species and genotypes

**Figure 2.**
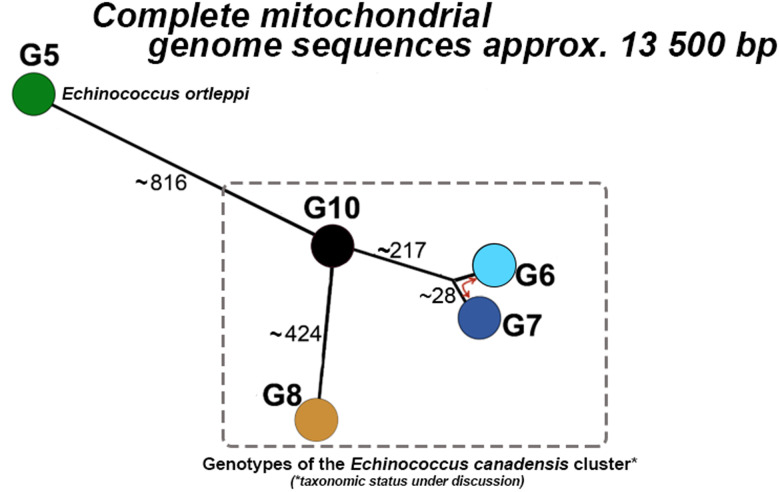
Schematic representation of the median-joining network based on representative complete mt genome sequences (~13 500 bp) of (i) genotypes G8 and G10 of the *E. canadensis* cluster – current study; (ii) *Echinococcus ortleppi* (G5) and genotypes G6 and G7 of the *E. canadensis* cluster – retrieved from GenBank. *Echinococcus ortleppi* is depicted in green, *E. canadensis* cluster genotype G8 in light brown, G10 in black and G6 and G7 in light and dark blue, respectively. The numbers on the lines represent the approximate number of mutations between the genotypes, indicating the minimum genetic distance between the closest nodes. GenBank accession numbers for the sequences are listed in Table S1 (dataset A).

The median-joining phylogenetic network of complete mt genomes for dataset A revealed that *Echinococcus ortleppi* (G5) is separated from G10 and G8 by more than 800 and 1200 mutations, respectively (schematic representation of genetic distances is shown in Fig. 2). As expected (see e.g. Moks *et al*., 2008; Nakao *et al*., 2013), genotype G10 is more closely related to genotypes G6/G7 (formerly camel and pig strains) than to the other cervid genotype G8 (~400 mutations between G8 and G10, and around 200 between G10 and G6/G7). The distance between genotypes G6 and G7 was ~25 mutations, in contrast to the vast distance between G8 and G10.

### Distinct patterns of variability within G8 and G10

**Figure 3.**
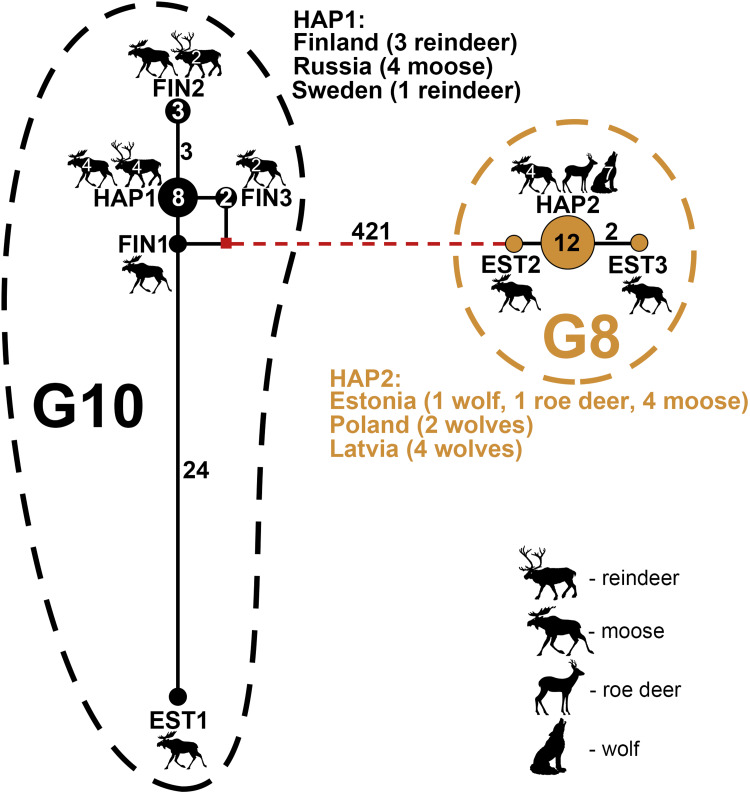
Median-joining network of genotypes G8 and G10 of the *E. canadensis* cluster (dataset B) based on complete mt genomes (13 550–13 552 bp). Genotype G8 haplotypes are depicted in light brown, G10 haplotypes are in black and median vectors as red rectangles. Numbers inside the circles represent the number of identical sequences within the respective haplotype; numbers on the lines represent the number of mutations. Note that the dashed line between the G10 and G8 haplotype clusters represents reduced edge-lengths. Haplotype names are designated as three-letter abbreviations (HAP, haplotypes representing samples originating from different countries; EST, Estonia; FIN, Finland). Black silhouettes of animals represent host species.

The phylogenetic network analysis of dataset B (complete mt genome sequences of *E. canadensis* G8 and G10 samples; *n* = 29) revealed a total of eight haplotypes, with G8 isolates represented by three haplotypes, and G10 isolates by five (Fig. 3). Within the G8 cluster, the majority of the samples (*n* = 12) were identical (haplotype HAP2), with HAP2 comprising samples from Estonia (*n* = 6), Latvia (*n* = 4) and Poland (*n* = 2), obtained from wolves, roe deer and moose. The remaining two G8 haplotypes originated from moose from Estonia (EST2 and EST3) and were separated from HAP2 by one and two mutations, respectively. The intra-genotypic variability within G10 appeared more complex than that of G8. Interestingly, the single G10 sample from Estonia from a moose was highly diverged, with 24–28 mutations separating it from the rest of the G10 samples from Sweden, Finland and Russia. The samples from the latter three countries revealed eight samples with an identical mt genome sequence; these samples originated from Sweden (reindeer, *n* = 1), Finland (reindeer, *n* = 3) and Russia (moose *n* = 4). The remaining samples from Finland were grouped into three separate haplotypes (FIN1–FIN3), with 1–3 mutations apart from HAP1. Similar to HAP1, haplotype FIN2 was identified from both reindeer and moose.

### Comparison of commonly applied mtDNA markers

An assessment of phylogenetic resolution provided by commonly used markers showed that while both the complete gene sequence and the widely used gene fragment of *cox*1 (1608 and 366 bp, respectively), the complete *nad*1 (894 bp) and its fragment (471 bp) and the complete 12S rRNA (726 bp) gene sequences are able to distinguish between G8 and G10 (schematic representation of genetic distances is shown in Fig. S1), they lack sufficient resolution to reveal detailed patterns of genetic variation within the two genotypes. An analysis based on a fragment of 12S rRNA (117 bp) revealed that G10 was indistinguishable from genotypes G6 and G7.

## Discussion

The complete mtDNA sequence data revealed a vast genetic distance (over 400 mutations) between the two cervid genotypes G8 and G10 (Fig. 2). Although previous studies have suggested the genetic distinctiveness of these genotypes (e.g. Nakao *et al*., 2006, 2013), this marked distance is somewhat surprising, first, given their biological and ecological similarities (Romig *et al*., 2017), and second, due to the species *E. ortleppi* (genotype G5) having only twice the distance to *E. canadensis* genotype G10 (~800 mutations). *Echinococcus ortleppi* has been firmly established as a distinct species and is predominantly transmitted through a cattle–dog cycle and has a distribution range distinct from the cervid genotypes (Romig *et al*., 2017). Hence, it would be expected that the mt genetic distance between *E. ortleppi* and *E. canadensis* G8/G10 would be several-fold greater than that between G8 and G10. This result could be explained by the divergence and subsequent evolution of the two mt lineages, G8 and G10, over an extended period of time. Whether this mt genetic distinctiveness has an impact on any phenotypic traits in these taxa warrants further investigations.

The finding of lower intraspecific variation within G8 and G10 could likely reflect the effect of limited sampling on the findings. Although the extent of genetic variation appeared to be lower than as reported for e.g. *E. granulosus s.s.* (Kinkar *et al*., 2018*a*, 2018*b*) or *E. granulosus s.l.* G7 (Laurimäe *et al*., 2018*b*), it was nonetheless observed that the intra-genotypic variation within G8 and G10 could be more complex than previously observed (e.g. Moks *et al*., 2008; Nakao *et al*., 2013). It is also worth noting that obtaining *Echinococcus* spp. samples is a labour-exhaustive task (typically involving slaughterhouse/abattoir surveys with veterinary supervision to inspect the internal organs of animals for possible *Echinococcus* cysts), particularly due to the low prevalence of *E. canadensis* G8 and G10 and its predominant perpetuation through a wildlife cycle. However, it could be hypothesized that the lower intraspecific variation could possibly be to some extent also explained by the small population size due to low prevalence and density of the parasite, as well as the rather restricted host spectrum (wild cervids and wolves) compared to other *Echinococcus* taxa e.g. *E. granulosus s.s.* G1 (Romig *et al*., 2017), and would lead to reduced mt genetic diversity over time (James and Eyre-Walker, 2020). Opposing theories have, however, stated a lack of such a correlation, possibly due to the stochasticity and high mutation rate of the mtDNA (Bazin *et al*., 2006). The lower genetic variation within G8 and G10 might also mirror local adaptation to these geographical regions. Although little is understood about the adaptive role of mutations in mtDNA in cestodes, it has been estimated that up to 23% of non-synonymous nucleotide substitutions in the mtDNA are fixed through adaptive evolution in mammals (James *et al*., 2016).

Interestingly, the pattern of mt genetic variation within G8 and G10 appeared to some extent reflect the dispersal pattern of their wild host animals. This is somewhat expected given that *E. canadensis* (G8/G10) is an obligatory parasite that relies on wild animals (wolves and moose) to maintain its life cycle. For example, genetic analyses of moose and wolf populations across Europe have suggested limited dispersal between Fennoscandia (Sweden, Finland) and continental Europe (including Estonia, Latvia and Poland), while dispersal within both regions, as well as between Finland and Russia (Arkhangelsk region) appears to be continuous (Niedziałkowska *et al*., 2016; Hindrikson *et al*., 2017). Indeed, results of the current study seem to suggest that samples of genotypes G8 from continental Europe are genetically similar, as evidenced by the shared haplotype (HAP2) from Estonia, Latvia and Poland; whereas within G10, the Fennoscandian (Finland, Sweden) and Russian samples showed close genetic similarity, while in contrast the sample from continental Europe (Estonia) appeared only distantly related to the rest of the G10 samples (Fig. 3). In the future it would be relevant to include more samples from other regions, including from North America and Far-East Asia, to determine the existence of other genetically divergent haplotypes/-groups within the two genotypes. This in-depth knowledge of genetic variation patterns within a parasite population could aid in the design of surveillance and control efforts in the future, should a need arise. Consequently, it could be hypothesized that were a new genetic variant or species of parasite (e.g. with higher pathogenicity and genetic variability) introduced into the wolf and moose populations in these areas, the parasite would likely be dispersed across vast distances, and depending on the phenotypic and biological characteristics of the new variants, could be a cause for concern for local wildlife, or even human health in some cases.

Finally, an assessment of phylogenetic resolution provided by the widely applied mtDNA markers highlighted the importance of selecting a genetic marker in accordance with the aim of the analysis, as also discussed in previous studies for other *Echinococcus* taxa (Kinkar *et al*., 2016; Laurimäe *et al*., 2018*b*). Complete mtDNA sequence datasets could be used to explore the genetic variation of *Echinococcus* taxa, whereas single mtDNA genes or gene fragments are better suited for species/genotype identification in instances where sequencing of complete mtDNA is not feasible.

The current study provides insight into the genetic variation of the elusive *E. canadensis* G8 and G10 genotypes based on complete mt genome data, highlighting marked mt genetic divergence between G8 and G10, and higher levels of intra-genotypic genetic variation than previously observed. Understanding the mt genetic composition of a species provides a baseline for future studies exploring the nature and extent of nuclear genomic variation, which might lead to an enhanced understanding of the molecular epidemiology of the *E. canadensis* cluster.

## Data Availability

All sequences are deposited in the GenBank (accession numbers OQ161094-OQ161122; Table 1).

## Supplementary material

For supplementary material accompanying this paper visit https://doi.org/10.1017/S0031182023000331. click here to view supplementary material
