# Supplementary material for: Exploring the genetic diversity of genotypes G8 and G10 of the Echinococcus canadensis cluster in Europe based on complete mitochondrial genomes (13 550–13 552 bp)
Source: Parasitology. 2023 Apr 3;150(7):631–7. doi: 10.1017/S0031182023000331 (PMC10260296; doi:10.1017/S0031182023000331)
Supplement: Supplementary file 1 [file S0031182023000331sup.zip › S0031182023000331sup002.pdf]

Table S1. Accession numbers, origin and dataset designation for sequences of the current study, as well as reference sequences retrieved from GenBank.

Dataset		Accession no	Species	Genotype	Country	Host	Publication	DOI	Sample ID:
A		KY766906	*E. ortleppi*	G5	India	buffalo	Kinkar et al. 2017	10.1016/j.meegid.2017.04.023	
A		KY766907	*E. ortleppi*	G5	India	buffalo	Kinkar et al. 2017		
A		KY766908	*E. ortleppi*	G5	India	buffalo	Kinkar et al. 2017		
A		MH300952	*E. canadensis cluster**	G6	Sudan	sheep	Laurimäe et al. 2018	10.1016/j.meegid.2018.06.016	
A		MH300939	*E. canadensis cluster*	G6	Sudan	camel	Laurimäe et al. 2018		
A		MH300950	*E. canadensis cluster*	G6	Sudan	camel	Laurimäe et al. 2018		
A		MH300940	*E. canadensis cluster*	G6	Sudan	sheep	Laurimäe et al. 2018		
A		MH300941	*E. canadensis cluster*	G6	Sudan	sheep	Laurimäe et al. 2018		
A		MH300942	*E. canadensis cluster*	G6	Sudan	sheep	Laurimäe et al. 2018		
A		MH300943	*E. canadensis cluster*	G6	Sudan	goat	Laurimäe et al. 2018		
A		MH300951	*E. canadensis cluster*	G6	Sudan	goat	Laurimäe et al. 2018		
A		MH300944	*E. canadensis cluster*	G6	Sudan	cattle	Laurimäe et al. 2018		
A		MH300945	*E. canadensis cluster*	G6	Sudan	cattle	Laurimäe et al. 2018		
A		MH300946	*E. canadensis cluster*	G6	Sudan	cattle	Laurimäe et al. 2018		
A		MH300947	*E. canadensis cluster*	G6	Sudan	sheep	Laurimäe et al. 2018		
A		MH300948	*E. canadensis cluster*	G6	Sudan	sheep	Laurimäe et al. 2018		
A		MH300949	*E. canadensis cluster*	G6	Sudan	goat	Laurimäe et al. 2018		
A		MH300936	*E. canadensis cluster*	G6	Kenya, Turkana	human	Laurimäe et al. 2018		
A		MH300938	*E. canadensis cluster*	G6	Kenya, Turkana	human	Laurimäe et al. 2018		
A		MH300937	*E. canadensis cluster*	G6	Kenya, Turkana	human	Laurimäe et al. 2018		
A		MH300931	*E. canadensis cluster*	G6	Iran	camel	Laurimäe et al. 2018		
A		MH300930	*E. canadensis cluster*	G6	Iran	camel	Laurimäe et al. 2018		
A		MH300932	*E. canadensis cluster*	G6	Iran	camel	Laurimäe et al. 2018		
A		MH300929	*E. canadensis cluster*	G6	Iran	camel	Laurimäe et al. 2018		
A		MH300933	*E. canadensis cluster*	G6	Argentina	goat	Laurimäe et al. 2018		
A		MH300934	*E. canadensis cluster*	G6	Argentina	goat	Laurimäe et al. 2018		
A		MH300935	*E. canadensis cluster*	G6	Argentina	goat	Laurimäe et al. 2018		
A		MH300953	*E. canadensis cluster*	G6	Mauritania	camel	Laurimäe et al. 2018		
A		MH300954	*E. canadensis cluster*	G6	Mauritania	camel	Laurimäe et al. 2018		
A		MH300969	*E. canadensis cluster*	G7	Argentina	pig	Laurimäe et al. 2018		
A		MH300984	*E. canadensis cluster*	G7	Serbia	pig	Laurimäe et al. 2018		
A		MH300972	*E. canadensis cluster*	G7	Mexico	pig	Laurimäe et al. 2018		
A		MH300979	*E. canadensis cluster*	G7	Mexico	pig	Laurimäe et al. 2018		
A		MH300978	*E. canadensis cluster*	G7	Mexico	pig	Laurimäe et al. 2018		
A		MH300974	*E. canadensis cluster*	G7	Mexico	pig	Laurimäe et al. 2018		
A		MH300981	*E. canadensis cluster*	G7	Mexico	pig	Laurimäe et al. 2018		
A		MH300975	*E. canadensis cluster*	G7	Mexico	pig	Laurimäe et al. 2018		
A		MH300976	*E. canadensis cluster*	G7	Mexico	pig	Laurimäe et al. 2018		
A		MH300977	*E. canadensis cluster*	G7	Mexico	pig	Laurimäe et al. 2018		
A		MH300980	*E. canadensis cluster*	G7	Mexico	pig	Laurimäe et al. 2018		
A		MH300973	*E. canadensis cluster*	G7	Mexico	pig	Laurimäe et al. 2018		
A		MH300983	*E. canadensis cluster*	G7	Romania	sheep	Laurimäe et al. 2018		
A		MH300982	*E. canadensis cluster*	G7	Romania	sheep	Laurimäe et al. 2018		
A		MH300985	*E. canadensis cluster*	G7	Spain	pig	Laurimäe et al. 2018		
A		MH300959	*E. canadensis cluster*	G7	Argentina	pig	Laurimäe et al. 2018		
A		MH300960	*E. canadensis cluster*	G7	Argentina	pig	Laurimäe et al. 2018		
A		MH300968	*E. canadensis cluster*	G7	Argentina	pig	Laurimäe et al. 2018		
A		MH300962	*E. canadensis cluster*	G7	Argentina	pig	Laurimäe et al. 2018		
A		MH300970	*E. canadensis cluster*	G7	Argentina	pig	Laurimäe et al. 2018		
A		MH300963	*E. canadensis cluster*	G7	Argentina	pig	Laurimäe et al. 2018		
A		MH300965	*E. canadensis cluster*	G7	Argentina	pig	Laurimäe et al. 2018		
A		MH300961	*E. canadensis cluster*	G7	Argentina	pig	Laurimäe et al. 2018		
A		MH300967	*E. canadensis cluster*	G7	Argentina	pig	Laurimäe et al. 2018		
A		MH300957	*E. canadensis cluster*	G7	Argentina	pig	Laurimäe et al. 2018		
A		MH300964	*E. canadensis cluster*	G7	Argentina	pig	Laurimäe et al. 2018		
A		MH300966	*E. canadensis cluster*	G7	Argentina	pig	Laurimäe et al. 2018		
A		MH300958	*E. canadensis cluster*	G7	Argentina	pig	Laurimäe et al. 2018		
A		MH300955	*E. canadensis cluster*	G7	Argentina	pig	Laurimäe et al. 2018		
A		MH300986	*E. canadensis cluster*	G7	France (Corsica)	pig	Laurimäe et al. 2018		
A		MH300995	*E. canadensis cluster*	G7	France (Corsica)	pig	Laurimäe et al. 2018		
A		MH300996	*E. canadensis cluster*	G7	France (Corsica)	pig	Laurimäe et al. 2018		
A		MH300997	*E. canadensis cluster*	G7	France (Corsica)	pig	Laurimäe et al. 2018		
A		MH300989	*E. canadensis cluster*	G7	France (Corsica)	pig	Laurimäe et al. 2018		
A		MH300987	*E. canadensis cluster*	G7	France (Corsica)	pig	Laurimäe et al. 2018		
A		MH300999	*E. canadensis cluster*	G7	France (Corsica)	pig	Laurimäe et al. 2018		
A		MH300998	*E. canadensis cluster*	G7	France (Corsica)	pig	Laurimäe et al. 2018		
A		MH300992	*E. canadensis cluster*	G7	France (Corsica)	pig	Laurimäe et al. 2018		
A		MH301002	*E. canadensis cluster*	G7	France (Corsica)	pig	Laurimäe et al. 2018		
A		MH300990	*E. canadensis cluster*	G7	France (Corsica)	pig	Laurimäe et al. 2018		
A		MH300991	*E. canadensis cluster*	G7	France (Corsica)	pig	Laurimäe et al. 2018		
A		MH300993	*E. canadensis cluster*	G7	France (Corsica)	pig	Laurimäe et al. 2018		
A		MH301000	*E. canadensis cluster*	G7	France (Corsica)	pig	Laurimäe et al. 2018		
A		MH301001	*E. canadensis cluster*	G7	France (Corsica)	pig	Laurimäe et al. 2018		
A		MH300994	*E. canadensis cluster*	G7	France (Corsica)	pig	Laurimäe et al. 2018		
A		MH300988	*E. canadensis cluster*	G7	France (Corsica)	pig	Laurimäe et al. 2018		
A		MH301003	*E. canadensis cluster*	G7	Poland	human	Laurimäe et al. 2018		
A		MH301007	*E. canadensis cluster*	G7	Poland	human	Laurimäe et al. 2018		
A		MH301005	*E. canadensis cluster*	G7	Poland	human	Laurimäe et al. 2018		
A		MH301022	*E. canadensis cluster*	G7	Ukraine	pig	Laurimäe et al. 2018		
A		MH301004	*E. canadensis cluster*	G7	Poland	pig	Laurimäe et al. 2018		
A		MH301020	*E. canadensis cluster*	G7	Lithuania	pig	Laurimäe et al. 2018		
A		MH301006	*E. canadensis cluster*	G7	Poland	pig	Laurimäe et al. 2018		
A		MH301021	*E. canadensis cluster*	G7	Ukraine	pig	Laurimäe et al. 2018		
A	B	OQ161094	*E. canadensis cluster*	G8	Latvia	wolf	current study		Sample1
A	B	OQ161095	*E. canadensis cluster*	G8	Latvia	wolf	current study		Sample2
A	B	OQ161096	*E. canadensis cluster*	G8	Latvia	wolf	current study		Sample3

A	B	OQ161097	*E. canadensis cluster*	G8	Latvia	wolf	current study			Sample4
A	B	OQ161098	*E. canadensis cluster*	G8	Poland	wolf	current study			Sample5
A	B	OQ161099	*E. canadensis cluster*	G8	Poland	wolf	current study			Sample6
A	B	OQ161100	*E. canadensis cluster*	G8	Estonia	wolf	current study			Sample7
A	B	OQ161101	*E. canadensis cluster*	G8	Estonia	roe deer	current study			Sample8
A	B	OQ161102	*E. canadensis cluster*	G8	Estonia	moose	current study			Sample9
A	B	OQ161103	*E. canadensis cluster*	G8	Estonia	moose	current study			Sample10
A	B	OQ161104	*E. canadensis cluster*	G8	Estonia	moose	current study			Sample11
A	B	OQ161105	*E. canadensis cluster*	G8	Estonia	moose	current study			Sample12
A	B	OQ161106	*E. canadensis cluster*	G8	Estonia	moose	current study			Sample13
A	B	OQ161107	*E. canadensis cluster*	G8	Estonia	moose	current study			Sample14
A	B	OQ161108	*E. canadensis cluster*	G10	Estonia	moose	current study			Sample15
A	B	OQ161109	*E. canadensis cluster*	G10	Finland	moose	current study			Sample16
A	B	OQ161110	*E. canadensis cluster*	G10	Finland	moose	current study			Sample17
A	B	OQ161111	*E. canadensis cluster*	G10	Finland	moose	current study			Sample18
A	B	OQ161112	*E. canadensis cluster*	G10	Finland	moose	current study			Sample19
A	B	OQ161113	*E. canadensis cluster*	G10	Finland	reindeer	current study			Sample20
A	B	OQ161114	*E. canadensis cluster*	G10	Finland	reindeer	current study			Sample21
A	B	OQ161115	*E. canadensis cluster*	G10	Finland	reindeer	current study			Sample22
A	B	OQ161116	*E. canadensis cluster*	G10	Finland	reindeer	current study			Sample23
A	B	OQ161117	*E. canadensis cluster*	G10	Finland	reindeer	current study			Sample24
A	B	OQ161118	*E. canadensis cluster*	G10	Russia (Arkhangelsk region)	moose	current study			Sample25
A	B	OQ161119	*E. canadensis cluster*	G10	Russia (Arkhangelsk region)	moose	current study			Sample26
A	B	OQ161120	*E. canadensis cluster*	G10	Russia (Arkhangelsk region)	moose	current study			Sample27
A	B	OQ161121	*E. canadensis cluster*	G10	Russia (Arkhangelsk region)	moose	current study			Sample28
A	B	OQ161122	*E. canadensis cluster*	G10	Sweden	reindeer	current study			Sample29

* - name and designation as according to Vuitton et al. 2020; DOI: https://doi.org/10.1051/parasite/2020024
