# Supplementary figures and images for: Exploring the genetic diversity of genotypes G8 and G10 of the Echinococcus canadensis cluster in Europe based on complete mitochondrial genomes (13 550–13 552 bp)
Source: Parasitology. 2023 Apr 3;150(7):631–7. doi: 10.1017/S0031182023000331 (PMC10260296; doi:10.1017/S0031182023000331)

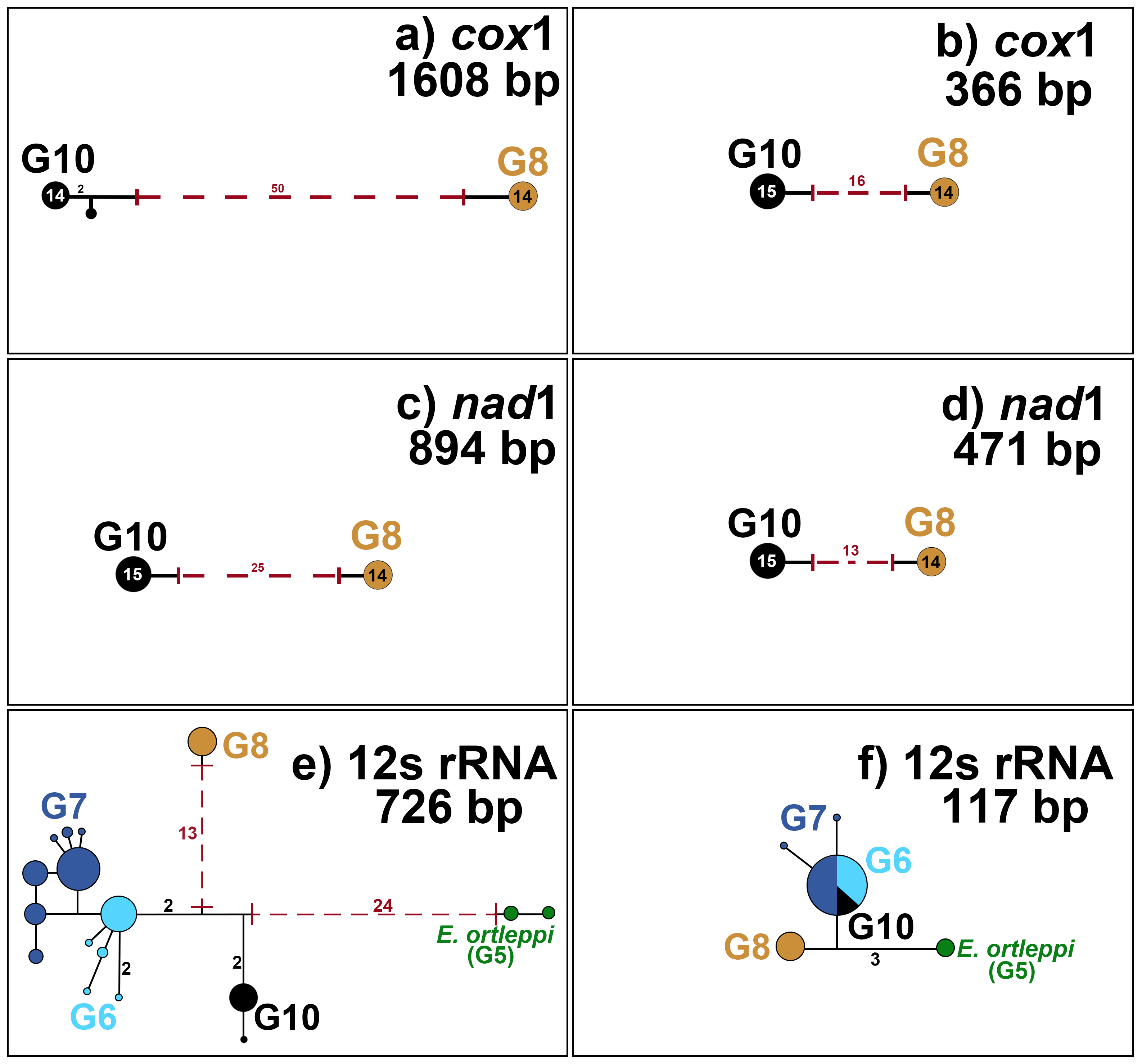

Supplement: Supplementary file 1 [file S0031182023000331sup.zip › S0031182023000331sup001.tif]
